# Objectively Measured Physical Activity Predicts Hip and Spine Bone Mineral Content in Children and Adolescents Ages 5–15 Years: Iowa Bone Development Study

**DOI:** 10.3389/fendo.2014.00112

**Published:** 2014-07-15

**Authors:** Kathleen F. Janz, Elena M. Letuchy, Shelby L. Francis, Kristen M. Metcalf, Trudy L. Burns, Steven M. Levy

**Affiliations:** ^1^Department of Health and Human Physiology, University of Iowa, Iowa City, IA, USA; ^2^Department of Epidemiology, University of Iowa, Iowa City, IA, USA; ^3^Department of Preventive and Community Dentistry, University of Iowa, Iowa City, IA, USA

**Keywords:** accelerometry, adolescence, childhood, dual-energy x-ray absorptiometry, exercise, mechanical loading, skeletal health

## Abstract

This study examined the association between physical activity (PA) and bone mineral content (BMC; gram) from middle childhood to middle adolescence and compared the impact of vigorous-intensity PA (VPA) over moderate- to vigorous-intensity PA (MVPA). Participants from the Iowa bone development study were examined at ages 5, 8, 11, 13, and 15 years (*n* = 369, 449, 452, 410, and 307, respectively). MVPA and VPA (minutes per day) were measured using ActiGraph accelerometers. Anthropometry was used to measure body size and somatic maturity. Spine BMC and hip BMC were measured via dual-energy x-ray absorptiometry. Sex-specific multi-level linear models were fit for spine BMC and hip BMC, adjusted for weight (kilogram), height (centimeter), linear age (year), non-linear age (year^2^), and maturity (pre peak height velocity vs. at/post peak height velocity). The interaction effects of PA × maturity and PA × age were tested. We also examined differences in spine BMC and hip BMC between the least (10th percentile) and most (90th percentile) active participants at each examination period. Results indicated that PA added to prediction of BMC throughout the 10-year follow-up, except MVPA, did not predict spine BMC in females. Maturity and age neither modify the PA effect for males nor females. At age 5, the males at the 90th percentile for VPA had 8.5% more hip BMC than males in the 10th percentile for VPA. At age 15, this difference was 2.0%. Females at age 5 in the 90th percentile for VPA had 6.1% more hip BMC than those in the 10th percentile for VPA. The age 15 difference was 1.8%. VPA was associated with BMC at weight-bearing skeletal sites from childhood to adolescence, and the effect was not modified by maturity or age. Our findings indicate the importance of early and sustained interventions that focus on VPA. Approaches focused on MVPA may be inadequate for optimal bone health, particularly for females.

## Introduction

Skeletal fractures associated with osteoporosis have significant health consequences including chronic pain, loss of function, and loss of independence (1). The economic cost of osteoporotic fractures is also high and growing, e.g., by 2025 in the U.S., fractures are projected to cost of $25.3 billion/year (2). Previous epidemiological studies have estimated that a 10% increase in peak bone mass may reduce osteoporotic fracture risk by 50% in post-menopausal women (3, 4). Because of this, prevention strategies during childhood and adolescence aimed at increasing peak bone mass are critical.

A promising method for preventing osteoporosis is participation in bone-strengthening physical activity (PA). The ability of PA to stimulate bone remodeling (osteogenic potential) is determined by the dynamic and odd nature of the load, magnitude of the load, rate at which the load is applied, and duration of the loading session. In general, activities that produce ground reaction forces (GRF) >1–2 times body weight or require significant muscle loading of the bones are more effective (5). Because of its importance in osteoporosis prevention (6), bone-strengthening PA has been included in the PA Guidelines for Americans, recommending that children and adolescents include these activities as part of their 60 min of daily PA on at least 3 days/week (7).

The beneficial effects of PA on bone health throughout the lifespan are evident (6). For example, a review of controlled trials of weight-bearing exercise and bone mineral content (BMC) in children and adolescents from 3 to 17 years (8) conducted by Hind and Burrows (9) found increases in BMC of 0.9–4.9% in prepubertal children, 1.1–5.5% in early pubertal children, and 0.3–1.9% in pubertal children. However, only one study examined spine BMC and hip BMC in children younger than age 7 and reported that PA did not increase BMC (10). The paucity of studies examining young children’s PA and BMC make it difficult to understand expected dose–response and therefore plan appropriate interventions. In addition, PA dose on BMC during intervention studies may not necessarily be generalizable to everyday physical activities that children and adolescents would voluntarily select. Therefore, the purpose of our study was to observationally and longitudinally examine the association between PA and BMC, including young children (age 5) and to compare the impact of vigorous-intensity PA (VPA) over moderate- to vigorous-intensity PA (MVPA) from ages 5 to 15.

## Materials and Methods

### Participants

Participants were recruited for the Iowa bone development study between 1998 and 2001 from a cohort of families participating in the Iowa fluoride study. The Iowa bone development study is a longitudinal study of bone health during childhood, adolescence, and young adulthood. Additional information about the study design and demographic characteristics of the participants have been described elsewhere (9, 11–13). This report focuses on data collected at ages 5, 8, 11, 13, and 15 years (*N* = 369, 449, 452, 410, and 307, respectively). To be included in the analyses, participants were required to have at least two measurements with at least one of those measurements occurring after age 8. Approval for this study was obtained from the University of Iowa Institutional Review Board for human subjects. Parents provided written informed consent and participants provided assent.

### Physical activity

Physical activity was measured via the ActiGraph activity monitor model 7164 at ages 5, 8, 11, and 13. Due to the unavailability of this ActiGraph model at the age 15 measurement, model GT1M was used. Previous research has shown a high correlation (*r* = 0.99) in movement counts data between the two monitors (14). Movement counts (a proxy for acceleration magnitude) were collected in 1-min epochs for ages 5, 8, 11, and 13 years and 5-s epochs for age 15. The 5-s epochs at age 15 were later reintegrated to 1-min epochs to maintain consistency with the earlier measurements. Participants at ages 5 and 8 were asked to wear the monitor during all waking hours for four consecutive days, including one weekend day. Using the Spearman–Brown prophecy formula, this amount of wear time provided an 82% reliability coefficient (15). Older children have previously been shown to have less stable intra-class correlation coefficients in activity monitored PA compared to younger children, indicating the need for an additional day of monitoring (16). Therefore, at ages 11, 13, and 15, the participants were asked to wear the monitor for five consecutive days, including both weekend days. To be included in the analyses, participants were required to have at least three valid days of monitor wear for each measurement period. A day was considered valid if the monitor was worn for at least 8 h. To reduce seasonal effects, PA was only measured during the autumn months.

The PA variables of interest were time spent in MVPA (minutes per day) and time spent in VPA (minutes per day). After comparing five independently developed sets of cut points on a sample of 5–15 years old, Trost and colleagues recommended that researchers use the Evenson cut points (17, 18) for children and adolescents. As specified by Evenson and colleagues, cut points were defined as ≥2296 counts/min for MVPA and ≥4012 counts/min for VPA. These cut points were evaluated using area-under-the-receiver operating characteristic (ROC-AUC) curve and have been shown to exhibit good classification accuracy separately (moderate ROC-AUC = 0.74; vigorous ROC-AUC = 0.84). When combined to MVPA, the cut points exhibited excellent classification accuracy (ROC-AUC = 0.90) (17).

### Bone mineral content

Bone mineral content (gram) of the lumbar spine and hip was determined using dual-energy x-ray absorptiometry (DXA) during clinical visits to the University of Iowa General Clinical Research Center by one of three trained technicians. Scans using Hologic QDR 2000 DXA (Hologic, Inc., Bedford, MA, USA) were conducted with software version 7.20B and pencil-beam mode at ages 5 and 8. When the participants were 11, 13, and 15, the Hologic QDR 4500 DXA (Delphi upgrade) with software version 12.3 and fan-beam mode was used. Software-specific global regions of interest (ROI) were used to designate the general boundaries of the spine and hip images. A review of the bone within the ROI box was confirmed by the operator and edited to ensure appropriate bone-edge detection. Quality control scans were performed daily using the Hologic spine phantom. To minimize operator-related variability, all measurements were conducted by one of three experienced technicians. The precision error for BMC measurements is low in our laboratory (coefficient of variation of <1% for quality control scans performed daily using the Hologic phantom). Translational equations for 4500 DXA measures to 2000 DXA measures were used to adjust for the differences between the two DXA machines. A separate study was conducted where 60 children (32 boys and 28 girls) ages 9–12 were scanned on each machine in random order during one clinical visit. The actual observations were closely aligned around the translational equation regression line, and the coefficient of determination (*R*^2^) for the 4500 DXA regressed on the 2000 DXA data was 0.98 [unpublished observation, linear regression for spine BMC: intercept = 2.57 (SE = 0.4), slope = 0.92 (SE = 0.02); hip BMC: intercept = 1.04 (SE = 0.4), slope = 0.993 (SE = 0.002)].

### Anthropometry

At the DXA visits, research nurses measured height (centimeter) and weight (kilogram). Height was measured using a Harpenden stadiometer (Holtain, UK), and weight was measured using a Healthometer physician’s scale (Continental, Bridgeview, IL, USA); both devices were routinely calibrated. Participants were measured while wearing indoor clothes, without shoes. At ages 11, 13, and 15 years, sitting height was also measured and used to estimate maturity offset [year from peak height velocity (PHV)] using predictive equations established by Mirwald and colleagues (19). These equations include age, sex, weight, height, sitting height, and leg length as predictors of years from PHV or somatic maturity. This method has been validated in white Canadian children and adolescents (*R*^2^ = 0.91–0.92, SEE = 0.49–0.50) (19). Somatic maturity was dichotomized as 0 (pre PHV, or premature) or 1 (at/post PHV, or mature).

### Statistical analysis

Descriptive statistics (means, SD, medians, and interquartile range) were calculated for the anthropometric, PA, and BMC characteristics of the participants. Student’s *t*-tests were used to examine sex differences. Sex-specific multi-level models (random- and fixed-effects) (SAS 9.2 MIXED procedure) were used to create BMC growth curves for individual participants (level 1) and to test the group effect of PA (level 2). This approach allowed us to include participants who missed measurement periods. In the multi-level models, the intercept and slope for age (at the time of the DXA scan) were specified as random effects and PROC MIXED estimated their variance–covariance parameters (20). Residual diagnostic plots were used to check the model assumptions and possible outliers. Time-varying predictors that changed over the multiple assessments included height (centimeter), weight (kilogram), linear age (year), non-linear age (year^2^) (to allow for non-linearity of growth), maturity (Pre PHV = 0; at/post PHV = 1), and either MVPA or VPA. Box–Cox transformations were used for MVPA and VPA variables due to skewed distributions. We tested the interaction effects of PA × maturity and PA × age. The Akaike information criterion (AIC) determined the fit of the models. Lower AIC values describe better fits. The differences in BMC between the least (10th percentile) and most (90th percentile) active children were estimated as predicted values from growth models with typical trajectories of covariates’ change over time.

## Results

### Participant characteristics

The participant characteristics are shown in Table 1. Males were significantly taller than females at ages 5, 8, 13, and 15 (*p* < 0.05) and significantly heavier than females at ages 8 and 15 (*p* < 0.05). Males participated in significantly more MVPA and VPA than females at every measurement period (*p* < 0.01). For males, the mean number of minutes of MVPA and VPA/day increased until age 11, then decreased thereafter. For females, the highest mean number of minutes of MVPA/day occurred at age 5 and decreased thereafter, but VPA increased from age 5 to 8, then decreased thereafter. There were no statistically significant differences in the mean number of minutes the ActiGraph was worn/day between males and females. Males had significantly more spine BMC than females at ages 5 and 8 (*p* < 0.05), but females had significantly more spine BMC than males at ages 11 and 13 (*p* < 0.05). At age 15, there was no significant difference in spine BMC between males and females. Males had significantly more hip BMC than females at ages 8, 13, and 15 (*p* < 0.05), but there were no significant differences in hip BMC between males and females at ages 5 and 11 (*p* > 0.05).

**Table 1:** **Characteristics of the participants by sex and age**.

	Age 5 years	Age 8 years	Age 11 years	Age 13 years	Age 15 years
Males	(*n* = 172)	(*n* = 215)	(*n* = 217)	(*n* = 205)	(*n* = 158)
Age (years)	5.2 (0.4)	8.7 (0.6)	11.2 (0.3)	13.3 (0.4)	15.4 (0.3)
Height (cm)	112.3 (5.8)*	134.7 (7.1)**	149.1 (7.6)	163.0 (9.5)**	175.2 (7.9)**
Weight (kg)	20.5 (3.6)	33.7 (9.4)*	45.3 (13.1)	58.2 (16.2)	70.4 (16.2)**
MVPA (min/day)	59.0 (23.7)**	64.2 (27.3)**	64.4 (28.5)**	50.5 (23.6)**	37.6 (19.4)**
VPA (min/day)	12.9 (9.4)**	17.9 (13.7)**	22.2 (15.6)**	16.1 (11.8)**	10.4 (10.2)**
Monitor wear/day (min)	731.0 (44.1)	749.3 (42.4)	743.1 (45.2)	745.9 (55.9)	731.7 (74.6)
Spine BMC (g)	16.1 (2.4)*	24.0 (3.9)**	30.1 (5.3)**	41.8 (11.0)**	60.1 (13.2)
Hip BMC (g)	7.1 (1.5)	13.1 (2.9)**	19.2 (4.6)	27.6 (7.5)**	37.9 (8.6)**

	**Median (IR)**	**Median (IR)**	**Median (IR)**	**Median (IR)**	**Median (IR)**

MVPA (min/day)	56.3 (43.5,74.5)	63.8 (44.8,82.3)	60.2 (42.8, 84.4)	46.6 (33.4, 65.8)	34.8 (22.8, 51.8)
VPA (min/day)	10.4 (6.8,16.5)	14.0 (8.5,22.8)	18.0 (10.8, 29.8)	13.5 (7.6, 22.3)	7.1 (3.3, 14.2)

**Females**	**(*n* = 197)**	**(*n* = 234)**	**(*n* = 235)**	**(*n* = 205)**	**(*n* = 149)**

Age (years)	5.3 (0.4)	8.7 (0.6)	11.2 (0.3)	13.2 (0.4)	15.3 (0.3)
Height (cm)	110.0 (5.4)	132.7 (6.7)	149.2 (7.5)	160.5 (6.6)	164.4 (6.4)
Weight (kg)	20.0 (3.8)	31.8 (8.5)	44.5 (12.3)	55.5 (14.0)	61.6 (14.3)
MVPA (min/day)	46.7 (19.9)	45.9 (20.6)	38.6 (18.5)	32.3 (18.0)	25.9 (16.6)
VPA (min/day)	9.9 (7.9)	11.8 (8.8)	10.5 (8.3)	9.3 (9.2)	6.8 (8.2)
Monitor wear/day (min)	731.8 (43.7)	743.4 (43.3)	741.8 (48.6)	752.2 (63.4)	740.4 (74.2)
Spine BMC (g)	15.5 (2.4)	22.9 (4.0)	32.0 (7.9)	47.3 (10.8)	57.7 (10.7)
Hip BMC (g)	6.9 (1.3)	12.2 (2.6)	18.6 (4.5)	25.8 (5.3)	29.2 (5.7)

	**Median (IR)**	**Median (IR)**	**Median (IR)**	**Median (IR)**	**Median (IR)**

MVPA (min/day)	43.5 (31.8, 59.0)	43.3 (30.3, 58.5)	35.2 (25.8, 48.8)	31.0 (18.8, 41.8)	23.4 (13.3, 37.2)
VPA (min/day)	7.8 (4.8, 13.8)	9.4 (5.5, 15.3)	8.4 (4.6, 13.8)	6.3 (3.3, 11.8)	4.2 (1.5, 9.5)

*Values are presented as mean (SD) and when skewed medians (IR)*.

*Untransformed MVPA and VPA minutes reported here; Box–Cox transformed values used in all inferential analyses*.

*MVPA, moderate and vigorous-intensity physical activity; VPA, vigorous-intensity physical activity; BMC, bone mineral content*.

*p < 0.05, **p < 0.01 Student’s *t*-test of males vs. females.

### Associations between MVPA, VPA, and BMC

Results for sex-specific multi-level models examining the effects of MVPA on BMC and VPA on BMC are shown in Tables 2–5. For males, both MVPA and VPA were significantly associated with spine BMC and hip BMC. The AIC were similar for both models (MVPA spine AIC = 5471.7, VPA spine AIC = 5474.0; MVPA hip AIC = 4382.1, VPA hip AIC = 4382.2). For females, MVPA predicted hip BMC but not spine BMC; however, VPA predicted both spine BMC and hip BMC. The model fit was significantly better for models with VPA than MVPA (MVPA spine AIC = 5495.8, VPA spine AIC = 5490.6; MVPA hip AIC = 3909.3, VPA hip AIC = 3902.3). While both maturity offset and age were statistically significant in all BMC models, PA × maturity and PA × age interaction effects were not significant. This was true for both PA intensity levels (MVPA and VPA). We therefore present models without interaction in Tables 2–5.

**Table 2:** **Fixed-effects of the multi-level models examining the association of MVPA with spine and hip BMC in males**.

Effect	β estimate	SE	*p*-Value
**SPINE**			
Intercept	−16.81	3.66	<0.0001
Age centered (years)	1.89	0.16	<0.0001
Age centered squared (year^2^)	0.26	0.02	<0.0001
Height (cm)	0.29	0.03	<0.0001
Weight (kg)	0.04	0.02	0.0591
Maturity (pre vs. at/post PHV)	8.52	0.56	<0.0001
MVPA (min/day)	0.16	0.05	0.0020
**HIP**			
Intercept	−12.98	2.14	<0.0001
Age centered (years)	1.34	0.10	<0.0001
Age centered squared (year^2^)	0.14	0.01	<0.0001
Height (cm)	0.17	0.02	<0.0001
Weight (kg)	0.11	0.01	<0.0001
Maturity (pre vs. at/post PHV)	3.91	0.30	<0.0001
MVPA (min/day)	0.12	0.03	<0.0001

*Box–Cox transformed values for MVPA*.

**Table 3:** **Fixed-effects of the multi-level models examining the association of MVPA with spine and hip BMC in females**.

Effect	β estimate	SE	*p*-Value
**SPINE**			
Intercept	−6.11	3.35	0.0692
Age centered (year)	2.54	0.12	<0.0001
Age centered squared (years^2^)	0.30	0.01	<0.0001
Height (cm)	0.20	0.03	<0.0001
Weight (kg)	0.18	0.02	<0.0001
Maturity (pre vs. at/post PHV)	3.85	0.39	<0.0001
MVPA (min/day)	0.06	0.05	0.2319
**HIP**			
Intercept	−24.38	1.83	<0.0001
Age centered (years)	0.42	0.06	<0.0001
Age centered squared (year^2^)	0.10	0.01	<0.0001
Height (cm)	0.24	0.01	<0.0001
Weight (kg)	0.13	0.01	<0.0001
Maturity (pre vs. at/post PHV)	1.78	0.16	<0.0001
MVPA (min/day)	0.06	0.02	0.0072

*Box–Cox transformed values for MVPA*.

**Table 4:** **Fixed-effects of the multi-level models examining the association of VPA with spine and hip BMC in males**.

Effect	β estimate	SE	*p*-Value
**SPINE**			
Intercept	−15.77	3.66	<0.0001
Age centered (years)	1.88	0.16	<0.0001
Age centered squared (year^2^)	0.25	0.02	<0.0001
Height (cm)	0.29	0.03	<0.0001
Weight (kg)	0.04	0.02	0.0825
Maturity (pre vs. at/post PHV)	8.59	0.56	<0.0001
VPA (min/day)	0.25	0.10	0.0178
**HIP**			
Intercept	−12.16	2.14	<0.0001
Age centered (years)	1.34	0.10	<0.0001
Age centered squared (year^2^)	0.14	0.01	<0.0001
Height (cm)	0.17	0.02	<0.0001
Weight (kg)	0.11	0.01	<0.0001
Maturity (pre vs. at/post PHV)	3.96	0.30	<0.0001
VPA (min/day)	0.23	0.06	0.0001

*Box–Cox transformed values for VPA*.

**Table 5:** **Fixed-effects of the multi-level models examining the association of VPA with spine and hip BMC in females**.

Effect	β estimate	SE	*p*-Value
**SPINE**			
Intercept	−6.30	3.34	0.0599
Age centered (years)	2.53	0.12	<0.0001
Age centered squared (year^2^)	0.30	0.01	<0.0001
Height (cm)	0.20	0.03	<0.0001
Weight (kg)	0.19	0.02	<0.0001
Maturity (pre vs. at/post PHV)	3.80	0.39	<0.0001
VPA (min/day)	0.22	0.10	0.0223
**HIP**			
Intercept	−24.30	1.83	<0.0001
Age centered (years)	0.41	0.06	<0.0001
Age centered squared (year^2^)	0.10	0.01	<0.0001
Height (cm)	0.24	0.01	<0.0001
Weight (kg)	0.13	0.01	<0.0001
Maturity (pre vs. at/post PHV)	1.75	0.16	<0.0001
VPA (min/day)	0.17	0.05	0.0004

*Box–Cox transformed values for VPA*.

**Figure 1:**
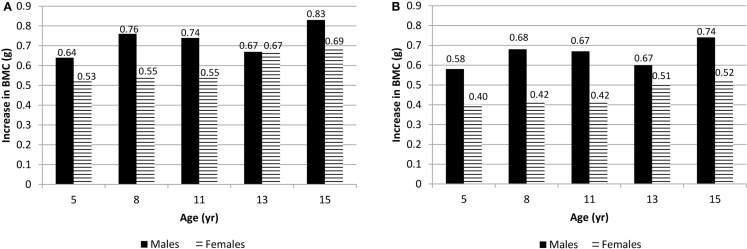
**(A)** Difference in spine BMC for high VPA (at the 90th percentile) over low VPA (at the 10th percentile) males and females at all measurement ages. **(B)** Difference in hip BMC for high VPA (90th percentile) over low VPA (10th percentile) males and females at all measurement ages. *p* < 0.05 at all ages.

At each measurement age, values were predicted for spine BMC and hip BMC for the 90th percentile (high VPA) and the 10th percentile (low VPA) (Figure 1). In these comparisons, weight, height, and maturity were set as the average values specific to sex and measurement age. Differences in BMC were statistically significant between high VPA and low VPA (*p* < 0.05) at all measurement ages. Greater percent differences in BMC were seen in younger participants with high vs. low VPA as compared to older participants because of both increase in BMC and decrease in VPA over study the period. For example, at age 5 years (15 years), males at the 90th percentile of VPA had 8.5% (2.0%) more hip BMC than those at the 10th percentile. For females, the differences were 6.1 and 1.8%, at ages 5 and 15 years, respectively.

## Discussion

This study examined the effect of PA on BMC from middle childhood to middle adolescence using two metabolic intensities, MVPA and VPA. Measurement of BMC at the spine and hip skeletal sites were chosen because they are clinically relevant for osteoporotic fractures. Spinal fractures can lead to chronic disabling pain (21), and ~20% of hip fracture patients require long-term nursing home care (6). The multi-level models, which we used fit each participant with an individual growth trajectory for spine BMC and hip BMC. This was important since the slope and intercept coefficients (level 1, not shown) varied across participants. This approach allowed us to identify the independent inter-group effects of PA on BMC while controlling for growth (age) and age-dependent covariates of height, weight, and maturity. With the exception of MVPA in the female spine BMC model, results showed that PA predicts BMC in males and females from ages 5 to 15 and the slope of the relationship between PA and BMC does not change across age groups or (somatic) maturity. The results also show that as PA decreases with aging, the effect size decreases. The percentage differences (~1–8%) that we report between high VPA participants (90th percentile) and low (10th percentile) are comparable to differences observed in targeted exercise interventions designed to increase BMC (10). For example, Gunter and colleagues reported that the 7- to 9-year-old children in their intervention group had 7.9% more BMC at the spine and 8.4% more BMC at the hip than the control group after a 7-month jumping intervention (22). Meyer and colleagues reported that the 6–7 and 11–12-year olds in their intervention group had 4.7% more bone mineral density (BMD) at the spine and 5.4% more BMD at the hip than the control group after a 9-month multi-component intervention including daily physical education with at least 10 min of jumping or strength training (23). Finally, McKay and colleagues reported a 1.9% increase in spine areal BMD and 3.2% increase in hip areal BMD in their third and fourth graders after participating in an 8-month jumping intervention twice/week during physical education classes. However, not all of the increases in areal BMD were significantly different from the increases seen in the control participants (24).

Similar reports from others (25–27), we found VPA to be a more consistent predictor of BMC than MVPA and, importantly, for the same duration of time, VPA would be expected to provide greater increases in BMC (when compared to MVPA). For females, the prediction slopes that we report would, on average, suggest that a 30-min/day increase in VPA would result in a 0.5-g increase in hip BMC (vs. 0.2 g for a similar increase in MVPA). For low active (10th percentile) 5-year-old females, this is an 8% increase in hip BMC associated with VPA (when compared to a 4% increase associated with MVPA). For males, increases of 0.7 and 0.5 g in hip BMC would be expected with a 30-min increase in VPA and MVPA, respectively. These values correspond to a 10% increase (VPA) vs. a 6% increase (MVPA) for 5-year-old low VPA males. Consistent with findings of Fuchs et al., MacKelvie et al., Heinonen et al., Stear et al., and Witzke and Snow, we also found greater increases in BMC at the hip when compared to the spine (28–32).

We have previously shown that the effect of PA at age 5 is sustained 3–6 years later (at age 8 and 11) but not at age 13 and 15 (33, 34) Our current report using a larger sample size and multi-level modeling, assesses acute effects of PA on BMC rather than sustained. The results suggest that what children and adolescents do in the present is associated with BMC. The lack of significance of the PA × maturity and PA × age interaction effects suggest that the BMC response to PA is consistent between 5 and 15 years of age. However, our data also show that the percent increases are greater when participants are more active. From a public health perspective, this finding is important since most young children are active and willing to engage in age-appropriate PA (when compared to adolescents) and therefore compliance with activity interventions is likely to be high. In addition, consistent with others (35–37), we have previously shown moderate tracking of activity behaviors in our cohort from ages 5 to 15 (38) suggesting that establishing a habit of bone-enhancing PA early in life is likely to continue through adolescence.

Limitations of our study include the use of a 1-min ActiGraph epoch, and a low minority, mostly white, convenience sample. It is possible that other, unmeasured factors could have contributed to differences in BMC, such as genetics or diet. Strengths of this report are the use of a longitudinal study design spanning 10 years, multi-level model to reduce confounding by growth and maturation, the clinically relevant outcomes of spine and hip BMC, and the use of an objective measure of PA. Although, we used a metabolic cut point to count minutes of PA, the ActiGraph accelerometer measures acceleration (meter per square second), which is proportional to the impact and muscular load forces acting on the skeletal system (22). Our placement of the ActiGraph at the waist provided a sensitive measure of weight-bearing PA but not swimming and cycling, activities thought to contribute little to bone loading. Recently, Deere and colleagues have shown positive associations between hip BMD and accelerometry-measured PA calibrated to mechanical load (gravitational force, g). Their findings indicated that only high impacts (>4.2 g) were associated with BMD. High impacts are, in general, associated with VPA.

In conclusion, we found that objectively measured VPA is associated with BMC at the spine and hip from childhood to adolescence. The magnitude of impact was greater when children were younger. Current public health approaches focusing on moderate PA, with an emphasis on reducing obesity, may be inadequate. Emphasizing VPA in young children, prior to the known decline in PA, may help to optimize peak BMC.

## Conflict of Interest Statement

The authors declare that the research was conducted in the absence of any commercial or financial relationships that could be construed as a potential conflict of interest.
